# Risk Factors Associated with Severe Underweight among Young Children Reporting to a Diarrhoea Treatment Facility in Bangladesh

**DOI:** 10.3329/jhpn.v28i5.6156

**Published:** 2010-10

**Authors:** Baitun Nahar, Tahmeed Ahmed, Kenneth H. Brown, Md. Iqbal Hossain

**Affiliations:** ^1^ ICDDR,B, GPO Box 128, Dhaka 1000, Bangladesh; ^2^ Institute of Mother and Child Health, Uppsala University, Sweden; ^3^ Department of Nutrition and Program in International and Community Nutrition, University of California Davis, USA; ^4^ Helen Keller International, Dakar, Senegal

**Keywords:** Body-weight, Case-control studies, Child, Child nutritional status, Infant, Infant nutritional status, Risk factors, Thinness, Bangladesh

## Abstract

Protein-energy malnutrition (PEM) is a serious health problem among young children in Bangladesh. PEM increases childhood morbidity and mortality. Information is needed on the major risk factors for PEM to assist with the design and targeting of appropriate prevention programmes. To compare the underlying characteristics of children, aged 6-24 months, with or without severe underweight, reporting to the Dhaka Hospital of ICDDR,B in Bangladesh, a case-control study was conducted among 507 children with weight-for-age z-score (WAZ) <-3 and 500 comparison children from the same communities with WAZ >-2.5. There were no significant differences between the groups in age [overall mean±standard deviation (SD) 12.6±4.1 months] or sex ratio (44% girls), area of residence, or year of enrollment. Results of logistic regression analysis revealed that severely-underweight children were more likely to have: undernourished mothers [body mass index (BMI) <18.5, adjusted odds ratio (AOR)=3.8, 95% confidence interval (CI) 2.6-5.4] who were aged <19 years (AOR=3.0, 95% CI 1.9-4.8) and completed <5 years of education (AOR=2.7, 95% CI 1.9-3.8), had a history of shorter duration of predominant breastfeeding (<4 months, AOR=2.3, 95% CI 1.6-3.3), discontinued breastfeeding (AOR=2.0, 95% CI 1.1-3.5), and had higher birth-order (>3 AOR=1.8, 95% CI 1.2-2.7); and fathers who were rickshaw-pullers or unskilled day-labourers (AOR=4.4; 95% CI 3.1-6.1) and completed <5 years of education (AOR=1.5; 95% CI 1.1-2.2), came from poorer families (monthly income of Tk <5,000, AOR=2.7, 95% CI 1.9-3.8). Parental education, economic and nutritional characteristics, child-feeding practices, and birth-order were important risk factors for severe underweight in this population, and these characteristics can be used for designing and targeting preventive intervention programmes.

## INTRODUCTION

Protein-energy malnutrition (PEM) is one of the most serious health problems in Bangladesh and other resource-poor countries where PEM accounts for more than 35% of deaths of children aged less than five years (under-five mortality) and 11% of the total burden of disease (1). Earlier reviews reported that severely-underweight children [weight-for-age z-score (WAZ) <-3], aged 6-59 months, had more than eight-fold increased mortality (2), and stunting, severe wasting, and intrauterine growth restriction together are responsible for 2·2 million deaths and 21% of disability-adjusted life-years (DALYs) for children aged less than five years (under-five children) (1). The latest national nutrition survey found that 29% of under-five children were moderately underweight (WAZ <-2 to -3), and 12% were severely underweight (WAZ <-3) (3).

The aetiology of childhood malnutrition is complex, involving interactions of the biological, cultural and socioeconomic factors. In most South Asian countries, poverty, high population density, low status of women, poor antenatal care, high rates of low birth weight, unfavourable child caring practices, and poor access to child healthcare are the underlying contributors to the development of PEM (4), although specific risk factors that can be used for targeting nutrition-intervention programmes have not been well-defined. Therefore, the objective of this study was to compare the characteristics of young children with or without severe underweight reporting to a diarrhoea treatment hospital in Bangladesh.

## MATERIALS AND METHODS

The study of young children, aged 6-24 months, who reported to the Dhaka Hospital of the International Centre for Diarrhoeal Disease Research, Bangladesh (ICDDR,B) during September 2005–June 2007, with or without severe underweight, used a case-control design. Cases with severe underweight were defined as children of either sex whose WAZ were <-3.0 with respect to the new growth standards of the World Health Organization (5) and who did not have any congenital anomalies that might have been responsible for their underweight. The controls were children from the same communities who reported to the hospital during the same period and whose WAZ were >-2.5 and length-for-age z-scores (LAZ) were >-3.0.

The Dhaka metropolitan area (1,500 sq km) has a total population of ∼11 million. Each year, the Dhaka Hospital of ICDDR,B provides care and treatment to over 100,000 patients with diarrhoea, with or without other associated health problems. The hospital also conducts research on enteric and other common infectious diseases and malnutrition and provides training on case management of diarrhoeal diseases and malnutrition and research methodology. Under-five children constitute about 60% of the total number of patients, and the majority (57%) of the patients come from poor socioeconomic communities in urban and peri-urban areas of Dhaka.

### Selection of cases and controls

Children enrolled in the study were recruited at the time of their discharge from inpatient treatment for diarrhoea and other acute illnesses. Cases were recruited from children who participated in a study of outpatient treatment of severely-underweight children (6). Children were selected as controls only if they resided in the same neighbourhoods as the cases. Each week/working day, potentially-eligible children who were ready for discharge from the hospital were weighed and measured, and the children who satisfied the entry criteria for cases and controls were invited to participate in the study. Verbal consent was obtained from a parent, usually the mother of the child.

### Collection of data

A research assistant interviewed the mothers using a pretested, structured questionnaire, and a medical doctor examined the children for signs of illness. Information recorded from the interviews included: age and sex of child, birth-order, number of total and under-five siblings, feeding and immunization history, type of residence and latrine, marital status of mother, monthly family income, and parental age, education, and occupation. The research assistant measured the children's nude weight, using a frequently-standardized digital scale with 10 g precision (Seca, model-345, Hamburg, Germany) and recumbent length to the nearest mm, using a calibrated, locally-constructed length board. The same research assistant also measured the weights and heights of mothers, using standard procedures (7). All measurements were taken twice, and the average was calculated; if they varied by more than 100 g for weight or 5 mm for length or height, a third measurement was taken, and the average of the nearest two measures was recorded. The study supervisor checked data forms daily. Anthropometric measurements were standardized during monthly refresher training sessions.

### Analysis of data

Data were entered using the SPSS software for Windows (version 11.5) (SPSS Inc., Chicago, IL, USA). For normally-distributed continuous variables, means were compared using unpaired *t*-tests after checking the equality of variance (Levene's test). For continuous variables that were not normally distributed, the Mann-Whitney U-test was performed. The differences in proportions were compared by the chi-square test or Fisher's exact test if the expected number in any cell was ≤5. A probability of less than 0.05 was considered statistically significant. The strength of association of selected risk factors for severe underweight was determined by estimating odds ratios (ORs) and their 95% confidence intervals (CIs). All independent variables, e.g. birth-order, number of siblings, socioeconomic status, parental characteristics, and child-feeding and immunization history, were analyzed initially in bivariate models and those that were significantly associated with severe underweight (dependent variable), biologically plausible, and not highly inter-correlated with each other (r<0.8) were included in multiple logistic regression models.

## RESULTS

In total, 1,007 children were studied, of whom 507 were cases and 500 were controls. Their overall mean±SD age was 12.6±4.1 months, and 44% were girls. There were no significant differences in the mean age or sex ratio in the two groups (Table 1). As dictated by the study design, the cases had significantly lower mean weight, length, WAZ, LAZ, and weight-for-length z-score (WLZ) than the control children (Table 1).

**Table 1.** Characteristics of cases (severely-underweight children) and controls

Variable	Case (n=507)	Control (n=500)	p value
Age (months) (mean±SD)	12.6±4.0	12.5±4.2	NS
Girls: no. (%)	221 (43.6)	220 (44.0)	NS
Residence*: no. (%)			
Demra	188 (37.1)	186 (37.2)	
Shabujbag	100 (19.7)	125 (25.0)	
Gulshan	117 (23.1)	107 (21.4)	
Mirpur	102 (20.1)	82 (16.4)	NS
Year of enrollment: no. (%)			
2005	87 (17.2)	70 (14.0)	
2006	223 (44.0)	216 (43.2)	
2007	197 (38.9)	214 (42.8)	NS
Child's body-weight (kg) (mean±SD)	6.03±0.86	8.60±1.31	<0.001
Child's length (cm) (mean±SD)	66.7±4.3	73.3±5.5	<0.001
Weigh-for-age z-score† (mean±SD)	-3.83±0.61	-0.83±0.83	<0.001
Weight-for-length z-score† (mean±SD)	-2.71±0.76	-0.55±1.12	<0.001
Length-for-age z-score† (mean±SD)	-3.56±0.99	-0.81±1.228	<0.001

*Name of the area (thana: area under one police station) of Dhaka city. Each area denotes children who came from that or surrounding 3-4 thanas of that area;

†In relation to the WHO 2006 standard; NS=Not significant; SD=Standard deviation; WHO=World Health Organization

The reported duration of predominant breastfeeding was significantly less among the severe- ly-underweight children [median 4.0 months; inter-quartile range (IQR) 1.3-6.0] than the control children (median 6.0, IQR 4.0-6.0, p<0.001), and the predominant breastfeeding rate until 5-6 months was significantly greater among the control children (68% vs 38% respectively, p<0.001) (Fig.). None of the children in either group was exclusively breastfed until six months. Approximately 15% of the severely-underweight children were never breastfed compared to <1% of the control children (p<0.001). In other bivariate analyses, increasing birth-order, higher number of total and under-five siblings, complete weaning, and absence of BCG, diphtheria, pertussis and tetanus (DPT)/polio, or measles immunization were significantly associated with severe underweight (Table 2 and 3). The mothers of malnourished children weighed less and were younger, shorter, less-educated, and more likely to be divorced or widowed and to work outside the home than the mothers of control children. Similarly, the fathers of malnourished children were younger, poorer, less-educated, and more likely to work at lower-paying jobs, such as rickshaw-pulling or day-labour.

**Fig.**
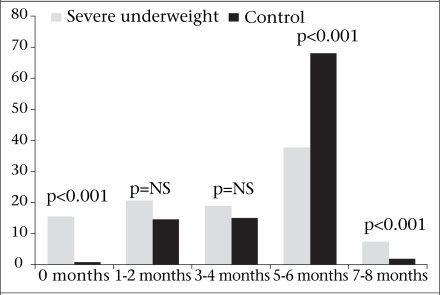
Percentage of severe underweight and control children who received predominant breast milk by age

NS=Not significant

**Table 2.** Parental and other family factors associated with presence of severe underweight

Factor	Case (n=507)	Control (n=500)	p value
Total number of children in family			
Median (inter-quartile range)	2 (1–2)	1 (1–2)	0.026
Number of under-five children in family			
Median (inter-quartile range)	1 (1–2)	1 (1–1)	0.002
Age of mother (years)*	23.5±5.5	24.3±4.4	0.013
Weight of mother (kg)*	42.20±6.86	49.37±8.53	<0.001
Height of mother (metre)*	1.48±0.05	1.50±0.05	<0.001
Body mass index of mother (kg/M^2^)*	19.36±2.84	21.99±3.55	<0.001
Education of mother (years)			
Median (inter-quartile range)	4 (0–5)	8 (5–10)	<0.001
Age (years) of father*	30.5±7.8	32.2±5.4	<0.001
Education of father (years)			
Median (inter-quartile range)	5 (0–8)	10 (5–12)	<0.001
Total family income per month (Tk†)	4,000	7,000	
Median (inter-quartile range)	(3,000-6,000)	(5,000-10,000)	<0.001

*Data are mean±SD if not mentioned otherwise;

†US$ 1=Tk 65—the average rate during the study period; SD=Standard deviation

**Table 3.** Attributes associated with the presence of severe underweight

Attribute	Case (n=507)		Control (n=500)		Odds ratio (95% CI)	p value
	No.	%	No.	%		
Birth-order (>3)	160	31.6	114	22.5	1.59 (1.19-2.12)	0.001
Predominant breastfeeding for <4 months	237	46.7	125	24.8	2.66 (2.02-3.51)	<0.001
Completely weaned	66	13.0	41	8.1	1.70 (1.11-2.62)	0.01
BCG not given	18	3.6	4	0.8	4.63 (1.51-18.91)	0.004†
DPT/polio not given (any dose)	14	2.8	4	0.8	3.57 (1.11-14.00)	0.029†
Measles vaccine not given (among >9 months old; n=380 cases and 406 controls)	94	24.7	67	16.5	1.54 (1.10-2.14)	0.005
Teen-aged mother (<19 years)	127	25	53	10.5	2.86 (1.99-4.12)	<0.001
Lighter mother (weight <44.5 kg)	348	71.3	160	31.6	5.39 (4.07-7.14)	<0.001
Shorter mother (height <1.5 metre)	307	62.8	233	46.0	1.79 (1.37-2.33)	<0.001
Undernourished mother (BMI <18.5)	213	43.7	82	16.2	4.03 (2.96-5.48)	<0.001
Illiterate or less-educated mother (<5 years of schooling)	384	75.7	186	37.5	5.20 (3.93-6.90)	<0.001
Mother working outside the home	92	18.1	55	10.8	1.82 (1.25-2.65)	0.001
Divorced or widowed mother	22	4.3	1	0.2	22.95 (3.67-949.09)	<0.001†
Younger father (age <25 years)	85	16.8	24	4.7	4.05 (2.47-6.68)	<0.001
Illiterate or less-educated father (<5 years of schooling)	308	60.7	114	28.4	4.93 (3.71-6.56)	<0.001
Father with lower-paying job (e.g. rickshaw-pulling or day-labour)	332	65.5	131	25.8	5.45 (4.12-7.20)	<0.001
Mothers having >2 children	123	24.3	89	17.6	1.50 (1.10-2.07)	0.01
Using unsanitary latrine	151	30	70	14	2.61 (1.88-3.62)	<0.001
Poor (monthly family income of Tk <5,000*)	384	75.7	186	37.5	5.20 (3.93-6.90)	<0.001

*US$ 1=Tk 65—the average rate during the study period;

†Fisher's exact test; BCG=Bacillus Calmette-Guérin; BMI=Body mass index; CI=Confidence interval; DPT=Diphtheria, pertussis and tetanus

The foregoing factors were included in a backward stepwise multiple logistic regression model. The regression model revealed that the severely-underweight children were more likely to have a father who had a lower-paying job [adjusted OR (AOR)=4.4, 95% CI 3.1-6.1) and less education (<5 years of education; AOR=1.5, 95% CI 1.1-2.2), a mother who was undernourished (BMI <18.5, AOR=3.7, 95% CI 2.6-5.4), younger (<19 years, AOR=3.0, 95% CI 1.9-4.8), and less-educated (AOR=2.7, 95% CI 1.9-3.8), and a family with low monthly income (monthly income of Tk <5,000, AOR=2.7, 95% CI 1.9-3.8) compared to the control children. Moreover, the malnourished children were more likely to have a short duration of predominant breastfeeding (<4 months, AOR=2.3, 95% CI 1.6-3.3), to be fully weaned (AOR=2.0, 95% CI 1.1-3.5), and to have a higher birth-order (>3, AOR=1.8, 95% CI 1.2-2.7).

## DISCUSSION

The results of our study showed that the major risk factors for severe underweight among 6-2-month- old Bangladeshi children hospitalized for the treatment of diarrhoea were related to parental education, employment, and income; child birth-order; and early child-feeding practices. Some of these factors may operate in common to increase the risk of undernutrition. Fathers of 65.5% of the severely-underweight children in our study were rickshaw-pullers or day-labourers with little or no formal education. Not only are these occupations the lowest-paid employment categories in Bangladesh but they also often result in erratic incomes, sometimes yielding in little or no earnings on a particular day. Although it is well-documented that level of parental education is positively associated with the nutritional status of their children (8), little information is available specifically on the importance of paternal education. Our finding that lower paternal education is a risk factor of severe underweight is in agreement with one other recently-published report (9) from Bangladesh, which found that education of father was significantly associated with both moderate and severe childhood stunting. Likewise, a study in Uganda found that both economic status and education of father were significantly associated with the nutritional status of children (10). Results of studies in South India showed that children of fathers who were day-labourers were ∼3 times more likely to be severely underweight (11).

As with other studies in Bangladesh (9,12) and Africa (13), the present study found that maternal malnutrition was an independent risk factor for severe underweight of children. Undernourished mothers often deliver low-birthweight babies (14), and their breastfeeding capacity may also be compromised (15–17); so, this association is not surprising.

The severely-underweight children in our study were also more likely to have illiterate or less-educated mothers (<5 years of schooling) as has been observed in previous studies in Bangladesh (9,12,18–22), and the underweight children were three times more likely to have teen-aged mothers than the better-nourished children. A previous report from Kenya also showed young maternal age to be a risk factor for severe PEM (OR=3.95, p<0.001) (23). Illiterate or less-educated and younger mothers usually have less knowledge of appropriate childrearing practices and optimal environmental and personal hygiene, and they generally have less status in the family (24,25), thereby rendering them less capable of providing adequate childcare and accessing formal health services.

Several previous studies examined the relationship between child birth-order and risk of malnutrition, although the results are inconsistent. One recently-published report from Bangladesh found a greater risk of stunting among higher birth-order children (9), and a study in South Africa found higher birth-order (>3) as a risk factor (OR=2.3, 95% CI 1.5-5.1) for all forms of severe PEM (marasmus, marasmic kwashiorkor, and kwashiorkor) (26). On the other hand, a study in Egypt did not find any relationship between birth-order and undernutrition (27).

Our finding of inadequate/improper breastfeeding as a risk factor corroborates the findings of several other studies in Bangladesh, which reported that inadequate breastfeeding, early supplementation of infant formula or cow's milk, and early introduction of semi-solid complementary foods were important risk factors for malnutrition (9,19,20,28). The hygienic and nutritional risks associated with bottle-feeding and artificial milks are well-known (29–31), and previous studies also found that breastfeeding had a significant and substantial impact on overall survival of undernourished children (32,33). It is also conceivable that the association with a shorter duration of predominant breastfeeding could be an example of reverse causality, whereby children who were ill and malnourished stopped nursing or were provided with other foods. Severely-malnourished children are more likely to have young mothers and to have been born prematurely or with a low birth- weight. Small, premature or malnourished infants tend to breastfeed less often and have a less vigorous suck (34). This produces less prolactin release and less production of breastmilk. Young mothers and mothers with low educational level may have less problem-solving skills, leading to an inability to support their infants when food supplies are limited, which, in turn, leads to malnutrition. The cross-sectional design of the present study does not allow us to distinguish between these possibilities.

**Table 4.** Factors associated with severe underweight: results of logistic regression model

Attribute	Adjusted odds ratio	95% CI of adjusted OR		p value
		Lower	Upper	
Father with lower job category (e.g. rickshaw-pulling or day-labour)	4.36	3.10	6.11	<0.001
Undernourished mother (BMI <18.5)	3.72	2.55	5.41	<0.001
Teen-aged mother (<19 years)	3.01	1.90	4.78	<0.001
Poor (monthly family income of Tk <5,000*)	2.71	1.94	3.78	<0.001
Illiterate or less-educated mother (<5 years of schooling)	2.67	1.86	3.84	<0.001
Predominant breastfeeding for <4 months	2.34	1.65	3.33	<0.001
Completely weaned	1.97	1.12	3.48	0.020
Shorter mother (height <1.5 metre)	1.93	1.39	2.69	<0.001
Higher birth-order (>3)	1.78	1.20	2.66	0.004
Illiterate or less-educated father (<5 years of schooling)	1.54	1.08	2.20	0.017
Constant	3.30	-	-	<0.001

Backward stepwise (likelihood ratio) logistic regression procedure. Variables removed from the equation: mothers having >2 children;

BCG not given;

mother working outside the home;

younger father (age <25 years);

divorced or widowed mother;

*US$ 1=Tk 65—the average rate during the study period;

BMI=Body mass index;

CI=Confidence interval;

OR=Odds ratio

The risk factors identified for severe underweight can be used, both to design and target preventive interventions. Several factors, such as those relating to infant-feeding practices and birth-order, are potentially modifiable; so, these can be used for designing intervention programmes. For examples, interventions can be developed to motivate behaviours that are more consistent with recommended feeding practices or to promote adoption of family-planning measures.

The causes of severe underweight are complex and involve the multiple dietary, environmental and other underlying socioeconomic factors, and carefully conceived, broad-based preventive programmes will be necessary to ameliorate the current situation.

## ACKNOWLEDGEMENTS

The study was funded by Sida-SAREC, Sweden and supported by ICDDR,B and its donors which provide unrestricted support to the Centre for its operations and research. Current donors providing unrestricted support include: Australian Agency for International Development (AusAID), Government of the People's Republic of Bangladesh, Canadian International Development Agency (CIDA), Embassy of the Kingdom of the Netherlands (EKN), Swedish International Development Cooperation Agency (Sida), and Department for International Development (DFID), UK. The authors gratefully acknowledge these donors for their support and commitment to the Centre's research efforts. The study was also supported by the Programme in International and Community Nutrition, University of California Davis and the Fogarty International Center (NIH Research Grant No. D43 TW01267).

The authors sincerely appreciate the statistical assistance of Dr. Rahman Azari of the Department of Statistics and the advice of Dr. Lucia L. Kaiser of the Department of Nutrition, University of California–Davis.

## References

[1] Black RE, Allen LH, Bhutta ZA, Caulfield LE, de Onis M, Ezzati M (2008). Maternal and child undernutrition: global and regional exposures and health consequences. Lancet.

[2] Pelletier DL, Frongillo EA, Schroeder DG, Habicht JP (1994). A methodology for estimating the contribution of malnutrition to child mortality in developing countries. J Nutr.

[3] National Institute of Population Research and Training (2009). Bangladesh demographic and health survey 2007.

[4] World Health Organization Report of the sixth meeting of South-East Asia Nutrition Research-cum-Action Network, Yangon, Myanmar, 6–8 February 2001.

[5] WHO Multicentre Growth Reference Study Group (2006). WHO child growth standards: length/height-for-age, weight-for-age, weight-for-length, weight-for-height and body mass index-for-age.

[6] Hossain MI, Nahar B, Ahmed T, Hamadani JD, Peerson JM, Brown KH (2009). Impact of community-based follow-up care, with or without food supplementation and/or psychosocial stimulation, on the recovery of severely underweight Bangladeshi children: a randomized intervention trial (abstract). Mal Med J.

[7] World Health Organization (1995). Physical status: the use and interpretation of anthropometry: report of a WHO expert committee.

[8] Cochrane SH, Leslie J, O'Hara DJ (1982). Parental education and child health: intracountry evidence. Health Policy Educ.

[9] Rahman A, Chowdhury S (2007). Determinants of chronic malnutrition among preschool children in Bangladesh. J Biosoc Sci.

[10] Kikafunda JK, Tumwine JK (2006). Diet and socio-economic factors and their association with the nutritional status of pre-school children in a low income suburb of Kampala city, Uganda. East Afr Med J.

[11] Saito K, Korzenik JR, Jekel JF, Bhattacharji S (1997). A case-control study of maternal knowledge of malnutrition and health-care-seeking attitudes in rural South India. Yale J Biol Med.

[12] Islam MA, Rahman MM, Mahalanabis D (1994). Maternal and socioeconomic factors and the risk of severe malnutrition in a child: a case-control study. Eur J Clin Nutr.

[13] Delpeuch F, Traissac P, Martin-Prével Y, Massamba JP, Maire B (2000). Economic crisis and malnutrition: socioeconomic determinants of anthropometric status of preschool children and their mothers in an African urban area. Public Health Nutr.

[14] Kramer MS (2003). The epidemiology of adverse pregnancy outcomes: an overview. J Nutr.

[15] Osteria TS (1982). Maternal nutrition, infant health, and subsequent fertility. Philipp J Nutr.

[16] Brown KH, Akhtar NA, Robertson AD, Ahmed MG (1986). Lactational capacity of marginally nourished mothers: relationships between maternal nutritional status and quantity and proximate composition of milk. Pediatrics.

[17] Brown KH, Robertson AD, Akhtar NA (1986). Lactational capacity of marginally nourished mothers: infants’ milk nutrient consumption and patterns of growth. Pediatrics.

[18] Henry FJ, Briend A, Fauveau V, Huttly SR, Yunus M, Chakraborty J (1992). The risk approach to intervention in severe malnutrition in rural Bangladesh. Am J Epidemiol.

[19] Henry FJ, Briend A, Fauveau V, Huttly SA, Yunus M, Chakraborty J (1993). Gender and age differentials in risk factors for childhood malnutrition in Bangladesh. Ann Epidemiol.

[20] Henry FJ, Briend A, Fauveau V, Huttly SR, Yunus M, Chakraborty J (1993). Risk factors for clinical marasmus: a case-control study of Bangladeshi children. Int J Epidemiol.

[21] Hossain MI, Yasmin R, Kabir I (1999). Nutritional and immunisation status, weaning practices and socio-economic conditions of under five children in three villages of Bangladesh. Indian J Public Health.

[22] Chisti MJ, Hossain MI, Malek MA, Faruque AS, Ahmed T, Salam MA (2007). Characteristics of severely malnourished under-five children hospitalized with diarrhoea, and their policy implications. Acta Paediatr.

[23] Ayaya SO, Esamai FO, Rotich J, Olwambula AR (2004). Socio-economic factors predisposing under five-year-old children to severe protein energy malnutrition at the Moi Teaching and Referral Hospital, Eldoret, Kenya. East Afr Med J.

[24] Goodburn E, Ebrahim GJ, Senapati S (1990). Strategies educated mothers use to ensure the health of their children. J Trop Pediatr.

[25] Radebe BZ, Brady P, Siziya S, Todd H (1996). Maternal risk factors for childhood malnutrition in the Mazowe district of Zimbabwe. Cent Afr J Med.

[26] Saloojee H, De Maayer T, Garenne ML, Kahn K (2007). What's new? Investigating risk factors for severe childhood malnutrition in a high HIV prevalence South African setting. Scand J Public Health.

[27] Hamid GA, El-Mougi M, El-Badrawy F, El-Mossallami MA (1978). An epidemiological study of energy protein malnutrition in a rural community in Egypt. J Egypt Med Assoc.

[28] Azad TMA, Bhuyan MAH, Khanam SA, Khan MSI, Rahim MA, Ahmed T (2006). A study of nourished vs severely-malnourished children admitted to a hospital: their feeding patterns and contributing factors (abstract). Abstracts book of the 8th Commonwealth Congress on Diarrhoea and Malnutrition, 6–8 February 2006, ICDDR,B.

[29] Jason JM, Nieburg P, Marks JS (1984). Mortality and infectious disease associated with infant-feeding practices in developing countries. Pediatrics.

[30] Brown KH, Black RE, Lopez de Romaña G, Creed de Kanashiro H (1989). Infant-feeding practices and their relationship with diarrheal and other diseases in Huascar (Lima), Peru. Pediatrics.

[31] Popkin BM, Adair L, Akin JS, Black R, Briscoe J, Flieger W (1990). Breast-feeding and diarrheal morbidity. Pediatrics.

[32] Briend A, Wojtyniak B, Rowland MG (1988). Breast feeding, nutritional state, and child survival in rural Bangladesh. Br Med J.

[33] Briend A, Bari A (1989). Breastfeeding improves survival, but not nutritional status, of 12–35 months old children in rural Bangladesh. Eur J Clin Nutr.

[34] Rikimaru T, Yartey JE, Taniguchi K, Kennedy DO, Nkrumah FK (1998). Risk factors for the prevalence of malnutrition among urban children in Ghana. J Nutr Sci Vitaminol (Tokyo).

